# Disease-specific and general health-related quality of life in newly diagnosed prostate cancer patients: the Pros-IT CNR study

**DOI:** 10.1186/s12955-018-0952-5

**Published:** 2018-06-13

**Authors:** Angelo Porreca, Marianna Noale, Walter Artibani, Pier Francesco Bassi, Filippo Bertoni, Sergio Bracarda, Giario Natale Conti, Renzo Corvò, Mauro Gacci, Pierpaolo Graziotti, Stefano Maria Magrini, Vincenzo Mirone, Rodolfo Montironi, Giovanni Muto, Stefano Pecoraro, Umberto Ricardi, Elvio Russi, Andrea Tubaro, Vittorina Zagonel, Gaetano Crepaldi, Stefania Maggi, the Pros-IT CNR study group, Gaetano Crepaldi, Stefania Maggi, Marianna Noale, Angelo Porreca, Walter Artibani, Pierfrancesco Bassi, Sergio Bracarda, Giario Natale Conti, Renzo Corvò, Pierpaolo Graziotti, Elvio Russi, Vincenzo Mirone, Rodolfo Montironi, Filippo Bertoni, Mauro Gacci, Stefano Maria Magrini, Giovanni Muto, Stefano Pecoraro, Umberto Ricardi, Andrea Tubaro, Vittorina Zagonel, Anna Rita Alitto, Enrica Ambrosi, Alessandro Antonelli, Cynthia Aristei, Michele Barbieri, Franco Bardari, Lilia Bardoscia, Salvina Barra, Sara Bartoncini, Umberto Basso, Carlotta Becherini, Rita Bellavita, Franco Bergamaschi, Stefania Berlingheri, Alfredo Berruti, Marco Borghesi, Roberto Bortolus, Valentina Borzillo, Davide Bosetti, Giuseppe Bove, Pierluigi Bove, Maurizio Brausi, Alessio Bruni, Giorgio Bruno, Eugenio Brunocilla, Alberto Buffoli, Michela Buglione, Consuelo Buttigliero, Giovanni Cacciamani, Michela Caldiroli, Giuseppe Cardo, Giorgio Carmignani, Giuseppe Carrieri, Emanuele Castelli, Elisabetta Castrezzati, Gianpiero Catalano, Susanna Cattarino, Francesco Catucci, Francolini Dario Cavallini, Ofelia Ceccarini, Antonio Celia, Francesco Chiancone, Tommaso Chini, Claudia Cianci, Antonio Cisternino, Devis Collura, Franco Corbella, Matteo Corinti, Paolo Corsi, Fiorenza Cortese, Luigi Corti, Cosimo de Nunzio, Olga Cristiano, Rolando M. D’Angelillo, Luigi Da Pozzo, Daniele D’agostino, Carolina D’Elia, Matteo Dandrea, Michele De Angelis, Paolo De Angelis, Ottavio De Cobelli, Bernardino De Concilio, Antonello De Lisa, Stefano De Luca, Agostina De Stefani, Chiara Lucrezia Deantoni, Esposti Claudio Degli, Anna Destito, Beatrice Detti, Nadia Di Muzio, Andrea Di Stasio, Calogero Di Stefano, Danilo Di Trapani, Giuseppe Difino, Sara Falivene, Giuseppe Farullo, Paolo Fedelini, Ilaria Ferrari, Francesco Ferrau, Matteo Ferro, Andrei Fodor, Francesco Fontanta, Francesco Francesca, Giulio Francolini, Paolo Frata, Giovanni Frezza, Pietro Gabriele, Maria Galeandro, Elisabetta Garibaldi, Pietro Giovanni Gennari, Alessandro Gentilucci, Alessandro Giacobbe, Laura Giussani, Giuseppe Giusti, Paolo Gontero, Alessia Guarneri, Cesare Guida, Alberto Gurioli, Dorijan Huqi, Ciro Imbimbo, Gianluca Ingrosso, Cinzia Iotti, Corrado Italia, Pierdaniele La Mattina, Enza Lamanna, Luciana Lastrucci, Grazia Lazzari, Fabiola Liberale, Giovanni Liguori, Roberto Lisi, Frank Lohr, Riccardo Lombardo, Jon A. J. Lovisolo, Giuseppe Mario Ludovico, Nicola Macchione, Francesca Maggio, Michele Malizia, Gianluca Manasse, Giovanni Mandoliti, Giovanna Mantini, Luigi Marafioti, Luisa Marciello, Alberto Mario Marconi, Antonietta Martilotta, Salvino Marzano, Stefano Masciullo, Gloria Maso, Adele Massenzo, Ercole Mazzeo, Luigi Mearini, Serena Medoro, Rosa Molè, Giorgio Monesi, Emanuele Montanari, Franco Montefiore, Giampaolo Montesi, Giuseppe Morgia, Gregorio Moro, Giorgio Muscas, Daniela Musio, Paolo Muto, Giovanni Muzzonigro, Giorgio Napodano, Carlo Luigi Augusto Negro, Mattia Nidini, Maria Ntreta, Marco Orsatti, Carmela Palazzolo, Isabella Palumbo, Alessandro Parisi, Paolo Parma, Nicola Pavan, Martina Pericolini, Francesco Pinto, Antonio Pistone, Valerio Pizzuti, Angelo Platania, Caterina Polli, Giorgio Pomara, Elisabetta Ponti, Antonio Benito Porcaro, Francesco Porpiglia, Dario Pugliese, Armin Pycha, Giuseppe Raguso, Andrea Rampini, Donato Franco Randone, Valentina Roboldi, Marco Roscigno, Maria Paola Ruggieri, Giuseppe Ruoppo, Roberto Sanseverino, Anna Santacaterina, Michele Santarsieri, Riccardo Santoni, Sarah Scagliarini, Giorgio Vittorio Scagliotti, Mauro Scanzi, Marcello Scarcia, Riccardo Schiavina, Alessandro Sciarra, Carmine Sciorio, Tindaro Scolaro, Salvatore Scuzzarella, Oscar Selvaggio, Armando Serao, Sergio Serni, Marco Andrea Signor, Mauro Silvani, Giovanni Silvano, Franco Silvestris, Claudio Simeone, Valeria Simone, Girolamo Spagnoletti, Matteo Giulio Spinelli, Luigi Squillace, Vincenzo Tombolini, Mariastella Toninelli, Luca Triggiani, Alberto Trinchieri, Luca Eolo Trodella, Lucio Trodella, Carlo Trombetta, Lidia Tronnolone, Marcello Tucci, Daniele Urzì, Riccardo Valdagni, Maurizio Valeriani, Maurizio Vanoli, Elisabetta Vitali, Alessandro Volpe, Stefano Zaramella, Guglielmo Zeccolini, Giampaolo Zini

**Affiliations:** 1 Policlinico di Abano Terme, Padova, Italy; 2 National Research Council (CNR), Neuroscience Institute, Aging Branch, Via Giustiniani 2, 35128 Padova, Italy; 3 Azienda Ospedaliera Universitaria Integrata di Verona, Verona, Italy; 4 Policlinico Universitario A. Gemelli, Università Cattolica del Sacro Cuore di Milano - Sede di Roma, Roma, Italy; 5 Prostate Group of AIRO - Italian Association for Radiation Oncology, Milano, Italy; 6 Ospedale San Donato, Arezzo, Italy; 7 Ospedale Sant’Anna, Como, Italy; 8 IRCCS San Martino-IST, Genova, Italy; 9 Università di Firenze, Firenze, Italy; 10 Ospedale S. Giuseppe, Milano, Italy; 11 Università di Brescia, Brescia, Italy; 12 Università degli Studi di Napoli Federico II, Napoli, Italy; 13 Università Politecnica delle Marche, Ancona, Italy; 14 Università Campus Bio-medico, Roma, Italy; 15 Malzoni Center, Avellino, Italy; 16 Università di Torino, Torino, Italy; 17 A.O. S. Croce e Carle, Cuneo, Italy; 18 Ospedale Sant’Andrea, Roma, Italy; 19 Istituto Oncologico Veneto IOV-IRCCS, Padova, Italy

**Keywords:** Prostate cancer, Quality of life, Diagnosis, Pros-IT CNR study

## Abstract

**Background:**

The National Research Council (CNR) prostate cancer monitoring project in Italy (Pros-IT CNR) is an observational, prospective, ongoing, multicentre study aiming to monitor a sample of Italian males diagnosed as new cases of prostate cancer. The present study aims to present data on the quality of life at time prostate cancer is diagnosed.

**Methods:**

One thousand seven hundred five patients were enrolled. Quality of life is evaluated at the time cancer was diagnosed and at subsequent assessments via the Italian version of the University of California Los Angeles-Prostate Cancer Index (UCLA-PCI) and the Short Form Health Survey (SF-12).

**Results:**

At diagnosis, lower scores on the physical component of the SF-12 were associated to older ages, obesity and the presence of 3+ moderate/severe comorbidities. Lower scores on the mental component were associated to younger ages, the presence of 3+ moderate/severe comorbidities and a T-score higher than one.

Urinary and bowel functions according to UCLA-PCI were generally good. Almost 5% of the sample reported using at least one safety pad daily to control urinary loss; less than 3% reported moderate/severe problems attributable to bowel functions, and sexual function was a moderate/severe problem for 26.7%. Diabetes, 3+ moderate/severe comorbidities, T2 or T3-T4 categories and a Gleason score of eight or more were significantly associated with lower sexual function scores at diagnosis.

**Conclusions:**

Data collected by the Pros-IT CNR study have clarified the baseline status of newly diagnosed prostate cancer patients. A comprehensive assessment of quality of life will allow to objectively evaluate outcomes of different profile of care.

## Background

Prostate cancer was the most common cancer diagnosed in men worldwide in 2015 [1]. With the exclusion of skin cancers, it represents 20% of all malignancies diagnosed in Italian males 50 years old or older [2]. Survival rates after a prostate cancer diagnosis continue to rise; approximately 89% of Italian patients are still alive 5 years after diagnosis, with North-western regions showing better rates with respect to Southern ones [2].

Clinical cancer researchers and oncologists recognize the importance of measuring survival and the clinical effects of treatments as well as patients’ quality of life in terms of subjective perceptions of symptoms, including physical, emotional and social functions [3, 4]. The increasing numbers of men with prostate cancer diagnoses and rising life expectancies underscore the importance of evaluating the quality of life of these patients [5, 6]. A number of studies have demonstrated that prostate cancer and its treatments affect physical and psychological health, as well as urinary, bowel and sexual function, with effects that seem to differ depending on the stage of the disease and the treatment being given [5, 7].

The National Research Council (CNR) prostate cancer monitoring project in Italy (Pros-IT CNR) is an ongoing study that is monitoring a sample population of Italian patients who were enrolled at the time they were diagnosed as new cases of prostate cancer. It aims to analyze the quality of life and general psychological and physical health parameters in real-world treatment situations during a 60 month study period. The current article reports on the health and quality of life registered at the study’s baseline when the patients were newly diagnosed with prostate cancer.

## Methods

### Study design

The Pros-IT CNR study design has been described elsewhere [8]. Briefly, the Pros-IT CNR is a multicenter, prospective study that aims to monitor the quality of life of a sample of Italian male patients 18 years and older who were diagnosed with biopsy-verified treatment-naïve prostate cancer after September 1, 2014.

Ninety-seven centers including 51 Urology, 39 Radiation Oncology and 7 Oncological facilities located throughout Italy were actively involved in the enrollment phase. The baseline questionnaires were administered at the time prostate cancer was diagnosed. Six follow-up evaluations 6, 12, 24, 36, 48 and 60 months after the original diagnostic assessment were scheduled for the patients.

### Ethics

The Pros-IT CNR study protocol was approved by the Ethics Committee of the clinical coordinating center located at the Sant’Anna Hospital (Como, Italy; register number 45/2014). It was also approved by the Ethics Committees of each of the other participating centers. The study was carried out in accordance with the principles of the Declaration of Helsinki; all the participants gave informed consent.

### Outcomes measures

Patients’ quality of life was evaluated using the validated Italian version of the University of California Los Angeles-Prostate Cancer Index (Italian UCLA-PCI; [9]) and the validated version of the Short Form Health Survey (SF-12 Standard v1 scale; [10]). Both questionnaires were recommended for use in men with prostate cancer by the authors of a recent systematic review [11]. The UCLA-PCI which received high ratings for its psychometric properties (content validity, internal consistency, construct validity and reproducibility), was recommended to evaluate health-related quality of life in prostate cancer patients. UCLA-PCI specifically evaluates urinary function and bother (UF, UB), bowel function and bother (BF, BB), and sexual function and bother (SF, SB). Scores range from 0 to 100, with higher score indicating better conditions.

SF-12 received high ratings for its criterion validity, construct validity, reproducibility, and interpretability; it was also recommended in view of its shortness [11]. The patients’ Physical and mental quality of life (Physical Component Summary (PCS) and Mental Component Summary (MCS), respectively) were evaluated using the SF-12 and possible scores range from 0 to 100, with 100 indicating best self-perceived health. Patients, who were originally evaluated at the time of diagnosis/enrollment, and are re-assessed at each of the appointments scheduled over the 60-month study period.

### Data collection

The participating centers identified eligible patients who were newly diagnosed with prostate cancer. After signing the informed consent form, a baseline Data Collection Form (DCF) was completed by the referring specialist using a web-platform that was specifically created for the study. The Italian version of the UCLA-PCI questionnaire was, instead, printed and completed by each patient privately, and then returned to the specialist who loaded the responses into the web-platform.

### Statistical analysis

The missing baseline data were analyzed without imputation of missing data.

Categorical variables are presented as numbers and percentages. Continuous variables are reported as means and standard deviations (SD) or medians and interquartile ranges for skewed variables. Normal distributions of continuous variables were tested using the Shapiro-Wilk test.

The patients’ overall quality of life, assessed using the SF-12 (PCS and MCS), and their quality of life linked to urinary, bowel and sexual function, assessed using the Italian version of the UCLA-PCI, were analyzed in relation to demographic characteristics, risk factors and disease-staging using a Generalized Linear Model (GLM) on rank-transformed data, adjusting for age at diagnosis.

Multivariable logistic regression models were defined, with outcomes the PCS and MCS SF-12 scores as well as the urinary, bowel and sexual functions of the UCLA-PCI, dichotomized according to the first quartile of their distribution (Q1). Each model was adjusted for age at diagnosis (years), education (lower secondary school diploma or less vs high school diploma or University degree), marital status (married or cohabitating vs widowed, divorced or single), geographical area of residence (northern regions of Italy vs central or southern regions), Body Mass Index (BMI; normal weight vs overweight or obesity), smoking status (current smoker vs former or never), diabetes mellitus, having three or more moderate/severe comorbidities, T stage (T1 vs T2 or T3-T4) and Gleason score at diagnosis (6 vs 3 + 4, 4 + 3, 8+).

All statistical tests were two-tailed, and *p*-values < 0.05 were considered statistically significant. All the analyses were performed using the SAS 9.4 statistical software.

## Results

One thousand seven hundred fifty-three patientswith a biopsy-verified prostate cancer were originally enrolled. Forty-eight protocol violations were registered in relation to inclusion criteria: diagnoses were formulated before September 1, 2014 for 35 patients and 13 were not naïve to prostate cancer treatments. Excluding those patients, our sample was made up of 1705 patients: 949 (55.7%) were enrolled in Urology, 717 (42.1%) in Radiation Oncology and 39 (2.3%) in Oncological Departments.

More than half of the participants were residing at the time of diagnosis in Northern Italy, about a quarter in Central Italy and the rest in Southern regions of the country. A “health migration” phenomenon was noted in these patients, as many travelled to centers located in the North to undergo diagnosis and/or treatment. In fact, 13.7 and 9% of patients residing in the South and Central areas, respectively, were enrolled at centers located in the North.

### Socio-demographic characteristics

The main socio-demographic data are presented in Table 1. The patients’ mean age at diagnosis was 68.9 ± 7.4 years. Almost 12% of the participants had a university degree, 36% had a high school diploma, and almost 30% had completed elementary school or had no study degree. Eighty-five percent of the participants were married or cohabiting. More than 90% of the participants were living with other members of their family such as a spouse and/or children. Approximately three-quarters were retired.

**Table 1:** Socio-demographic characteristics and anamnestic data of the participants of the Pros-IT CNR study at the time they were diagnosed with prostate cancer

	*n* = 1705
Socio-demographic characteristics at diagnosis	
Age at diagnosis, years	
mean ± SD	68.9 ± 7.4
min, max	43, 86
Education, n (%)	
University degree	103 (12.0)
High school diploma	596 (35.6)
Lower secondary school diploma	393 (23.5)
Elementary license or less	485 (28.9)
Marital status, n (%)	
Married or cohabiting	1442 (84.9)
Widowed	98 (5.8)
Separated, divorced or single	159 (9.4)
Living arrangements, n (%)	
With spouse and/or children	1535 (90.3)
Alone	165 (9.7)
Work condition, n (%)	
Retired	1263 (74.7)
Still working	398 (23.6)
Unemployed	29 (1.7)
Anamnestic data at diagnosis	
BMI, n (%)	
Under/normal weight (< 25 kg/m^2^)	568 (34.2)
Overweight (25–29.9 kg/m^2^)	832 (50.1)
Obesity (≥30 kg/m^2^)	260 (15.7)
Smoking status, n (%)	
Current smoker	230 (13.8)
Former smoker	688 (41.3)
Never smoker	747 (44.9)
Family history of prostate cancer, n (%)	286 (17.0)
Family history of breast cancer, n (%)	80 (5.8)
Family history of ovarian cancer, n (%)	25 (2.1)
Diabetes mellitus, n (%)	263 (15.5)
Patients reporting moderate, severe or extremely severe impairment for individual CIRS items, n (%)	
Cardiac	322 (19.0)
Hypertension	17 (1.0)
Vascular, haematological	464 (27.3)
Respiratory	61 (3.6)
Eye, ear, nose and throat	146 (8.6)
Upper gastrointestinal	236 (13.9)
Lower gastrointestinal	88 (5.2)
Hepatic	110 (6.5)
Renal	86 (5.1)
Other genitourinary	51 (3.0)
Musculoskeletal, integumentary	82 (3.0)
Neurological, excluding dementia	174 (10.4)
Endocrine, metabolic	83 (4.9)
Psychiatric, behavioural	31 (1.8)

*SD* Standard Deviation, *CIRS* Cumulative Illness Rating Scale

### Anamnestic data

More than half of the patients were overweight and had a BMI between 25 and 29.9 kg/m^2^ (Table 1). Almost 14% declared that they were current smokers, while 41% were former smokers.

Seventeen percent of the patients reported having a family history of prostate cancer; 5.8 and 2.1%, respectively, reported family breast and ovarian cancer history. The mean age at the diagnosis of prostate cancer in the participants with a family history of the disease was significantly lower than that in those without one (66.8 ± 8.3 vs 69.3 ± 7.2, *p* < 0.0001).

Two hundred sixty three patients (15.5%) declared that they had diabetes mellitus. Four hundred sixty-four of the patients (27.3%) reported having moderate, severe or extremely severe diseases, as defined by the Cumulative Illness Rating Scale (CIRS; [12]), of the vascular, lymphatic or hematopoietic system; 322 (19.0%) referred having a disease of the cardiac system, 236 (13.9%) of the gastrointestinal apparatus and 174 (10.3%) of the neurological system, excluding dementia.

At enrollment, more than 70% of the participants were taking at least one medication; the median number of drugs assumed was three (interquartile range IQ 1–4). Precisely 53.7% were taking drugs for the circulatory system, 27% of the participants were receiving antithrombotic agents, 25.4% were medication for the digestive system and metabolism (16.4% for acidosis, 10.6% hypoglycemic drugs). About one quarter of the enrolled patients (22.6%) were taking urological drugs for lower urinary tract symptoms or for erectile dysfunction.

### Diagnosis

**Fig. 1:**
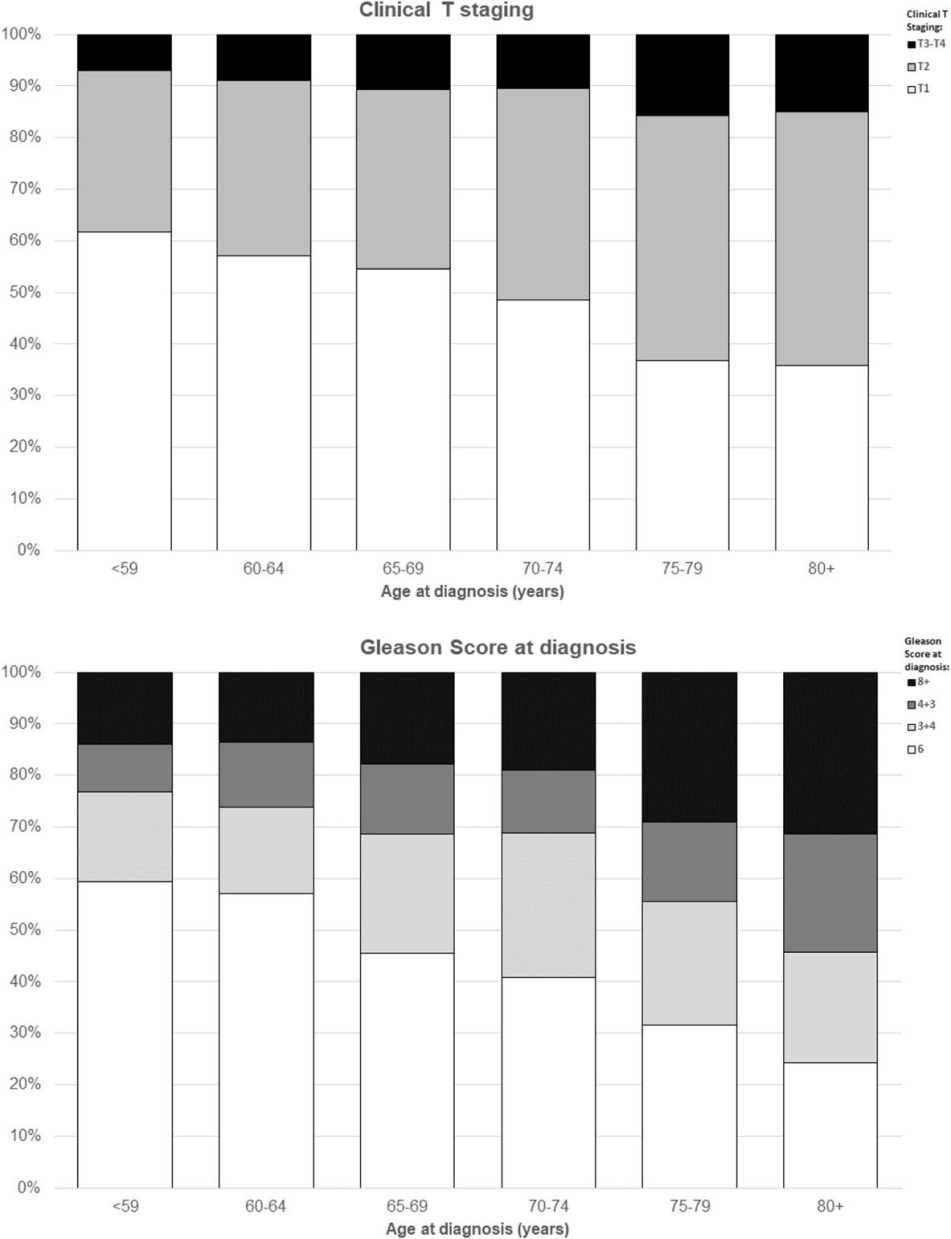
Clinical T staging (**a**) and Gleason score (**b**) of the participants of the Pros-IT CNR study by age classes at diagnosis

The median prostate-specific antigen (PSA) level at diagnosis was 7.2 ng/mL (IQ 5.2–10.6). Approximately half of the study participants had a T1 clinical stage (786, 48%), 38.6 and 11.4% had a T2 or T3-T4clinical stage, respectively. The Gleason score for prostate biopsy tissue was six for 718 patients (42.8%), 3 + 4 for 381 (22.7%), 4 + 3 for 233 (13.9%) and 8+ for 349 patients (20.8%). The association of age at diagnosis with both the clinical T stage and the Gleason score was significant (*p* < 0.0001 for trend; Fig. 1a, b).

### Quality of life: SF-12

Complete responses to the SF-12 were available for 1664 participants (data was missing for 2.4% of the participants). The mean PCS value for the entire study population was 51.6 ± 7.5; the mean MCS value was 49.3 ± 9.7. While mean PCS scores tended to be lower in the oldest patients (*p* < 0.0001), the mean MCS scores tended to be higher in the oldest patients (*p* = 0.0059).

Table 2 outlines the mean PCS and MCS values at diagnosis analyzed together with other characteristics of the participants. The characteristics associated with lower PCS scores in the multivariable logistic regression model were age (Odds Ratio OR 1.06 for each year of age, 95% Confidence Interval CI 1.04–1.08, *p* < 0.0001), obesity (OR 1.84, 95% CI 1.27–2.65, *p* = 0.0012), the presence of three or more moderate/severe comorbidities (OR 2.75, 95% CI 2.01–3.76, p < 0.0001) and a Gleason score at diagnosis of 8+ (OR = 1.44, 95% CI 1.02–2.05, *p* = 0.0401). Living in Southern regions of Italy and being widowed or single were also associated with lower PCS scores in the multivariable model (OR = 1.69, 95% CI 1.23–2.33, *p* = 0.0013 and OR = 1.42, 95% CI 1.02–1.98, p = 0.040, respectively).

**Table 2:** Demographic data and responses to the physical (PCS) and mental components (MCS) of the SF-12 of the participants of the Pros-IT CNR study at the time they were diagnosed with prostate cancer

	PCS*	p-value	MCS*	p-value
Age at diagnosis (years)		< 0.0001^§^		0.0058^§^
< 60	54.4 ± 6.2		47.3 ± 9.9	
60–64	52.8 ± 6.6		48.5 ± 9.7	
65–69	52.2 ± 6.8		50.2 ± 9.3	
70–74	51.1 ± 7.7		49.5 ± 10.1	
75–79	50.1 ± 8.0		49.9 ± 9.3	
80+	48.1 ± 10.2		49.9 ± 9.0	
Education		0.4460		0.5036
University degree	52.7 ± 6.6		49.6 ± 9.4	
High school diploma	52.1 ± 7.2		49.0 ± 9.5	
Lower secondary school diploma	51.2 ± 7.9		49.0 ± 9.8	
Elementary license or less	51.0 ± 7.9		50.0 ± 9.8	
Marital status		0.5575		0.4830
Married or cohabiting	51.8 ± 7.4		49.5 ± 9.4	
Widowed	51.1 ± 7.7		49.1 ± 10.2	
Separated, divorced or single	51.1 ± 8.0		48.0 ± 11.0	
Geographical area of residence		0.0002		0.0991
North Italy	51.7 ± 7.5^a^		49.9 ± 9.6	
Central Italy	52.3 ± 7.2^b^		49.0 ± 9.7	
Southern Italy	50.4 ± 7.8^ab^		48.7 ± 9.8	
BMI		< 0.0001		0.3796
Under/normal weight (< 25 kg/m^2^)	52.0 ± 7.5^a^		49.1 ± 9.7	
Overweight (25–29.9 kg/m^2^)	52.0 ± 7.1^b^		49.5 ± 9.5	
Obesity (≥30 kg/m^2^)	49.7 ± 8.4^ab^		49.7 ± 9.9	
Smoking status		0.1576		0.4419
Non-smoker or former	51.6 ± 7.6		49.4 ± 9.6	
Current smoker	51.9 ± 7.2		48.4 ± 10.1	
Diabetes mellitus		< 0.0001		0.1894
No	52.0 ± 7.3		49.4 ± 9.7	
Yes	49.6 ± 8.4		49.0 ± 9.6	
Number of moderate/severe comorbidities (according to CIRS)		< 0.0001		< 0.0001
0–2	52.5 ± 6.7		49.8 ± 9.4	
3+	47.0 ± 9.9		46.8 ± 10.4	
T staging at diagnosis		0.8226		< 0.0001
T1	52.0 ± 7.2		50.4 ± 9.3^ab^	
T2	51.6 ± 7.6		48.7 ± 9.6^a^	
T3 or T4	51.1 ± 7.9		48.6 ± 10.1^b^	
Gleason score at diagnosis		0.3409		0.1599
6	52.1 ± 7.0		49.6 ± 9.3	
3 + 4	51.9 ± 7.3		50.0 ± 9.5	
4 + 3	51.6 ± 7.8		48.6 ± 10.3	
8+	50.7 ± 8.3		48.7 ± 10.1	

^*^mean ± SD

^§^p-value from test for trend

^a, b^significant post-hoc (*p* < 0.05 adjusting for age at diagnosis)

The characteristics associated with lower MCS scores in the multivariable logistic model were younger age (OR = 0.97, 95% CI 0.96–0.98, p = 0.0012), the presence of three or more moderate/severe comorbidities (OR 1.95, 95% CI 1.42–2.70, *p* < 0.0001), a T-score at diagnosis that was higher than T1 (T2 vs T1 OR 1.51, 95% CI 1.15–1.98, *p* = 0.0029; T3-T4 vs T1 OR 1.62, 95% CI 1.06–2.48, *p* = 0.0253).

### Quality of life: UCLA-PCI

Complete responses to the UCLA-PCI were available for 1645 participants (3.5% missing data). At the time prostate cancer was diagnosed, urinary function was good (93.2 ± 15.7) and urinary bother scores were low (88.5 ± 23.3). Four point 9 % of the study participants reported using at least one safety pad daily to control urinary loss; the increase with age was not significant (*p* = 0.1943): the percent ranged from 3.2% in the patients younger than 65 to 5.8, 5.3 and 5.4% in the patients between 65 and 69, 70–74 and 75-older, respectively. The use of a daily safety pad to control urinary loss was significantly associated with lower urinary bother scores (45.7 ± 29.3 vs 90.7 ± 20.7, p < 0.0001).

Bowel function and bother scores on the UCLA-PCI were generally good (93.6 ± 13.2 and 93.3 ± 18.3, respectively), and less than 3% of the participants reported a moderate or severe problem attributable to bowel function. The mean sexual function and bother scores were 48.6 ± 32.2 and 64.1 ± 35.0, respectively. Twenty-six point 7 % of the participants declared that their sexual function was a moderate/large problem: the percentages ranged from 22.9 to 23.8% to 29.8 and 30.5% in the patients younger than 65, between 65 and 69, 70–74 or 75 or older respectively (*p* = 0.0044).

**Fig. 2:**
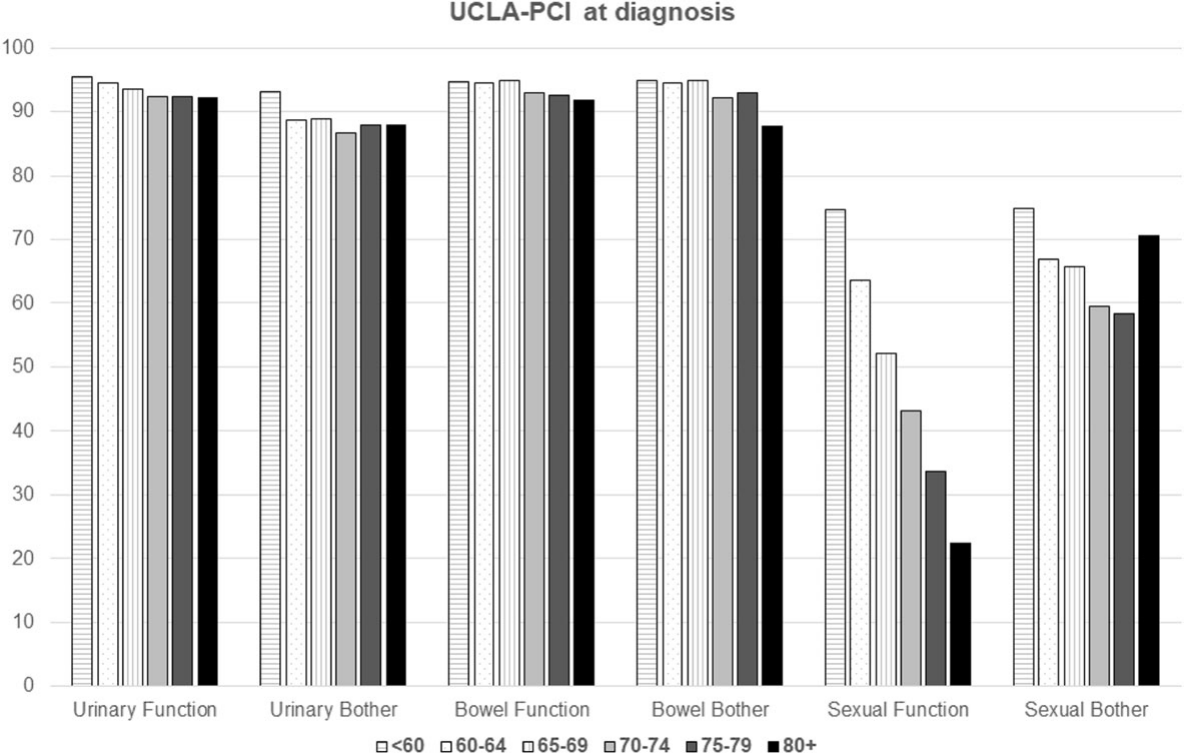
Mean responses regarding urinary, bowel and sexual function and bother (UCLA-PCI) of the participants of the Pros-IT CNR study by age classes at diagnosis

Age was the main characteristic associated with prostate cancer scores (Fig. 2; Table 3), also in the multivariable logistic regression models; the *p*-values for trend with increasing age were statistically significant for every health aspect evaluated by UCLA-PCI.

**Table 3:** Demographic data and responses regarding urinary, bowel and sexual function and bother to the UCLA-PCI of the participants of the Pros-IT CNR study at the time they were diagnosed with prostate cancer

	Urinary Function*	*p*-value	Urinary Bother*	*p*-value	Bowel Function*	*p*-value	Bowel Bother*	*p*-value	Sexual Function*	*p*-value	Sexual Bother*	*p*-value
Age at diagnosis		0.0015^§^		0.0030^§^		0.0058^§^		0.0167^§^		< 0.0001^§^		< 0.0001^§^
< 60 years	95.5 ± 12.4		93.3 ± 18.1		94.7 ± 12.4		94.9 ± 17.5		74.3 ± 25.0		74.8 ± 34.6	
60–64	94.3 ± 14.2		88.7 ± 23.7		94.5 ± 13.4		94.3 ± 15.7		63.2 ± 27.8		66.9 ± 34.6	
65–69	93.3 ± 16.3		88.9 ± 23.5		94.7 ± 11.4		94.6 ± 16.0		51.2 ± 30.4		65.3 ± 32.8	
70–74	92.2 ± 16.6		86.9 ± 24.5		92.9 ± 13.7		92.1 ± 19.6		43.1 ± 30.2		59.5 ± 35.1	
75–79	92.3 ± 16.3		87.5 ± 24.2		92.8 ± 13.9		93.3 ± 18.9		33.2 ± 29.5		58.5 ± 36.4	
80+	92.2 ± 16.7		88.0 ± 23.9		91.9 ± 16.2		87.1 ± 25.1		23.0 ± 25.3		70.3 ± 33.6	
Education		0.8255		0.7833		0.3867		0.1411		0.2339		0.4686
University degree	94.3 ± 14.2		89.3 ± 22.1		95 ± 11.2		93.1 ± 17.2		56.8 ± 32.6		67.7 ± 35.3	
High school diploma	93.6 ± 15.4		88.2 ± 23.8		93.4 ± 13		92.8 ± 18.7		52.2 ± 31.8		63.6 ± 35.2	
Lower secondary school diploma	92.8 ± 15.7		88.9 ± 23.5		94.4 ± 12.4		94.5 ± 16.9		49.6 ± 32.5		66.2 ± 34.3	
Elementary or less	92.4 ± 16.8		88.3 ± 23.4		92.8 ± 14.7		92.9 ± 19.3		39.9 ± 30.5		61.8 ± 35.2	
Marital status		0.1720		0.6739		0.0158		0.6490		0.5433		0.6904
Married/cohabiting	93.4 ± 15.6		88.7 ± 23.2		93.9 ± 13.1^a^		93.5 ± 18.1		49.1 ± 32.2		63.9 ± 35.1	
Widowed	91.7 ± 15		88.7 ± 22.9		92.7 ± 14.2		91.1 ± 21.9		40.6 ± 29.9		63.3 ± 34.5	
Separated, divorced, single	91.9 ± 17.1		87.1 ± 25.1		91.5 ± 13.9^a^		93 ± 18.3		48.4 ± 32.7		66.6 ± 34.8	
Geographical residence		0.0792		0.0988		0.1680		0.2818		0.0651		< 0.0001
North Italy	93.2 ± 15.3		88.3 ± 23.2		93.5 ± 12.8		93.1 ± 18.3		47.5 ± 32.3		67.8 ± 34.1^ab^	
Central Italy	92.7 ± 16.5		90.2 ± 22.1		94.7 ± 12.3		94.2 ± 17.6		51.4 ± 31.3		58.8 ± 35.3^a^	
South Italy	92.5 ± 16		86.9 ± 25.5		93 ± 14.9		92.8 ± 19.1		46.7 ± 33.2		60 ± 36.3^b^	
BMI		0.8219		0.3962		0.0290		0.6373		0.0012		0.3423
Under/normal weight (< 25 kg/m^2^)	93 ± 16.2		88.7 ± 23.9		94 ± 12.6^a^		93.3 ± 18.3		50.3 ± 32^a^		65.3 ± 34.8	
Overweight (25–29.9 kg/m^2^)	93.1 ± 15.6		88.7 ± 22.9		94.1 ± 12.5^b^		93.6 ± 17.6		48.9 ± 32.3^b^		64.1 ± 34.8	
Obesity (≥30 kg/m^2^)	93.3 ± 15.1		87.4 ± 23.7		91 ± 16.5^ab^		91.8 ± 21.0		43.4 ± 32^ab^		61.8 ± 36.1	
Smoking status		0.3011		0.2375		0.5201		0.8061		0.6522		0.9697
Non-smoker or former	92.9 ± 16		88.1 ± 23.9		93.4 ± 13.5		93.1 ± 18.6		47.8 ± 32.2		63.8 ± 35.2	
Current smoker	94.4 ± 14.3		91.1 ± 20.4		94.7 ± 11.3		93.9 ± 16.6		53.6 ± 31.2		66.1 ± 34.0	
Diabetes mellitus		0.3837		0.2607		0.6216		0.0585		< 0.0001		0.6154
No	93.3 ± 15.7		88.9 ± 23.0		93.8 ± 12.9		93.7 ± 17.7		50.5 ± 32.0		64.5 ± 34.9	
Yes	92.6 ± 15.9		86.9 ± 25.0		92.9 ± 14.6		91.1 ± 21.4		38.2 ± 31.0		62.0 ± 35.7	
N. of moderate/severe comorbidities (CIRS)		0.0774		0.0072		0.0001		< 0.0001		< 0.0001		0.1873
0–2	93.4 ± 15.5		89.4 ± 22.3		94.2 ± 12.5		94.5 ± 16.3		50.8 ± 31.9		64.9 ± 34.9	
3+	91.9 ± 16.6		83.8 ± 27.8		90.3 ± 16.3		86.5 ± 26.1		35.7 ± 30.7		60.2 ± 35.4	
T staging at diagnosis		0.1070		0.0133		0.2658		0.5184		< 0.0001		0.4077
T1	94.2 ± 14		90.3 ± 22		94.6 ± 12.2		94 ± 17.2		55.7 ± 30.6^a^		66.6 ± 33.8	
T2	92.8 ± 16.2		87.2 ± 24.4		93.6 ± 13.0		93.0 ± 18.3		43.5 ± 31.7^b^		62.8 ± 34.8	
T3 or T4	90.7 ± 19		86.4 ± 24.3		93.5 ± 13.6		93.2 ± 19.5		37.1 ± 32.3^ab^		63.6 ± 36.9	
Gleason score at diagnosis		0.6653		0.2894		0.8231		0.5100		< 0.0001		0.5015
6	93.9 ± 14.4		90.1 ± 21.6		94.3 ± 12.2		94.3 ± 16.6		54.8 ± 30.3		66.0 ± 34.1	
3 + 4	93.3 ± 16.4		88.9 ± 23.3		93.8 ± 12.5		92.8 ± 18.3		48.8 ± 31.7		62.7 ± 35.5	
4 + 3	93.7 ± 13.4		86.7 ± 25.0		93.4 ± 13.1		93.8 ± 17.0		44.9 ± 32.5		60.6 ± 35.9	
8+	91.6 ± 18.5		87.1 ± 24.8		93 ± 14.7		92.1 ± 21.2		39 ± 33.3		64.5 ± 35.7	

^*^mean ± SD

^§^p-value from test for trend

^a, b^significant post-hoc (p < 0.05 adjusting for age at diagnosis

Variables significantly associated with lower scores on sexual function in the multivariable models were age (OR 1.10, 95% CI 1.08–1.13, *p* < 0.0001), diabetes (OR = 1.40, 95% CI 1.01–1.96, *p* = 0.0485), three or more moderate/severe comorbidities according to CIRS (OR 1.55, 95% CI 1.11–2.16, *p* = 0.0103), T2 or T3-T4 at diagnosis (OR = 1.42, 95% CI 1.06–1.89, *p* = 0.0185, and OR = 1.75, 95% CI 1.14–2.69, *p* = 0.0093, vs T1, respectively) and a Gleason score of eight or more (OR = 2.03, 95% CI 1.42–2.92, *p* = 0.0001).

## Discussion

The PROS-IT CNR Study allows to assess the quality of life of males diagnosed as new cases of prostate cancer in Italy. More than half of the patients reside in Northern Italy, a fact that is linked to the geography of the centers, which all voluntarily agreed to participate in the study. Half, in fact, are located in the North; approximately a fourth are located in Central Italy and the rest in the Southern part of the country. According to the last report of the Italian Association of Cancer Registry (AIRTUM), the standardized incidence of prostate cancer was inferior in the Southern and Central regions with respect to that in the Northern ones (68 and 85.7 vs 99.8 per 100,000 men) [2]. Official records also show that while the Southern part of Italy is characterized by a lower incidence rate of prostate cancer, it nonetheless also registers a shorter survival rate. Our study will provide evidence on potential delay in the diagnosis of prostate cancer in South Italy, which could explain these epidemiological trends.

The patients participating in the Pros-IT CNR study were found to be characterized by a higher education with respect to data referring to the general population of males over 75 reported by the Italian Statistics Institute (ISTAT) [13]. While 12% of the men enrolled in our study had a university degree, only 7% of the general population did so. While 30% of the participants completed grade school or had no official schooling, 48.6% of the general male elderly population did so. The differences in educational status of the participants and the general population, seem to suggest that socio-economic characteristics might be associated to the risk of prostate cancer in the Italian population. We cannot however exclude a selection bias as more highly educated males may have agreed to participate in the study.

The mean score physical SF-12 component score was 51.7, which was higher than that described by the ISTAT in males between 65 and 74 (48.4) or those over 75 (41.5). The scores on the emotional-psychological SF-12 component in the participants were consistent with those reported by the ISTAT in males between 65 and 74 and slightly higher than those calculated for men over 75 [13]. These results disagree to some extent with what has been reported by other studies. For example, both the investigators of the ProtecT trial and a review on prostate cancer and health-related quality of life, reported scores on the two SF-12 components in just diagnosed patients that were consistent with those in the population at large [5, 7]. Moreover, according to other studies, just diagnosed with prostate cancer patients had lower scores on the emotional-psychological component with the respect to those in the general population [14, 15]. Again, these results could be explained by the higher level of education of the participants in our sample compared to that of the general population, a factor that is usually associated to an overall better physical and emotional health.

The scores on the physical component and thus concerning the perception of physical health were worse, in our study, in the older age-groups, while those concerning emotional-psychological status tended to be worse in the youngest age-group, suggesting, just as has been point out in other studies, that a certain amount of psyco-emotional adjustment takes place with aging [16, 17].

It is interesting that worse emotional-psychological component scores were associated to worse T classes at diagnosis regardless of age or other confounding factors such as comorbidities. Likewise, worse T or Gleason scores at diagnosis were associated to worse scores on sexual function, although in some studies men with localized prostate cancer reported more sexual problems with respect to same-age peers without cancer [3].

Approximately 5% of the patients included in our study declared that they used at least one safety pad daily to control urinary loss. That percentage did not change in the older patients, but it did when there were other urinary disturbances. This baseline finding is of primary relevance when post-treatment continence is being defined and evaluated.

The Pros-IT CNR study has several strengths, including its multidisciplinary approach and its prospective design. The study’s longitudinal design that foresees monitoring the participants for 60 months from the time of diagnosis, will allow to evaluate the disease’s evolution over time and the patients quality of life. One of the study’s limitations instead is connected to the fact that centres were involved on a voluntary basis and a selection bias cannot be excluded.

## Conclusions

The importance of the results presented here is twofold: they draw a profile of the general state of health and the subjective perception of quality of life of patients who have just been diagnosed with prostate cancer. Moreover, they underscore the patients’ characteristics at diagnosis that are relevant for appreciating the variations over time of their quality of life. More detailed knowledge about patients’ pre-treatment status and perception of health and quality of life will be essential to evaluate their response to treatment and to permit us to compare our data with those reported by other studies.

## Abbreviations

AIRTUM: Italian Association of Cancer Registry
BMI: Body Mass Index
CI: Confidence Interval
CIRS: Cumulative Illness Rating Scale
DCF: Data Collection Form
GLM: Generalized Linear Model
IQ: Interquartile range
ISTAT: Italian Statistics Institute
MCS: Mental Component Summary
OR: Odds Ratio
PCS: Physical Component Summary
PSA: Prostate-specific antigen
SD: Standard Deviation
SF-12: Short Form Health Survey
UCLA-PCI: University of California Los Angeles-Prostate Cancer Index
